# Multi-omic analyses of m5C readers reveal their characteristics and immunotherapeutic proficiency

**DOI:** 10.1038/s41598-024-52110-7

**Published:** 2024-01-18

**Authors:** Rui Xu, Yue Wang, Ye Kuang

**Affiliations:** 1 Department of Medical Laboratory, Yan’An Hospital of Kunming City, Kunming, Yunnan Province China; 2 Department of Endocrinology, The First Affiliated Hospital of Dalian Medical University, Dalian, Liaoning Province China; 3 Department of Development Planning, International Medical Opening-up Pilot Zone (China), Fangchenggang, Guangxi Province China

**Keywords:** Cancer, Computational biology and bioinformatics

## Abstract

5-methylcytosine (m5C) is a post-transcriptional RNA modification identified, m5C readers can specifically identify and bind to m5C. ALYREF and YBX1 as members of m5C readers that have garnered increasing attention in cancer research. However, comprehensive analysis of their molecular functions across pancancer are lacking. Using the TCGA and GTEx databases, we investigated the expression levels and prognostic values of ALYREF and YBX1. Additionally, we assessed the tumor microenvironment, immune checkpoint-related genes, immunomodulators, Tumor Immune Dysfunction and Exclusion (TIDE) score and drug resistance of ALYREF and YBX1. Gene Ontology (GO), Kyoto Encyclopedia of Genes and Genomes (KEGG), and Gene Set Enrichment Analysis (GSEA) analyses were performed to investigate the potential functions associated with m5C readers and coexpressed genes. Aberrant expression of ALYREF and YBX1 was observed and positively associated with prognosis in KIRP, LGG and LIHC. Furthermore, the expression levels of ALYREF and YBX1 were significantly correlated with immune infiltration of the tumor microenvironment and immune-related modulators. Last, our analysis revealed significant correlations between ALYREF, YBX1 and eIFs. Our study provides a substantial understanding of m5C readers and the intricate relationship between ALYREF, YBX1, eIFs, and mRNA dynamics. Through multidimensional analysis of immune infiltration and drug sensitivity/resistance in ALYREF and YBX1, we propose a possibility for combined modality therapy utilizing m5C readers.

## Introduction

In the untranslated regions (UTRs) of mRNA transcripts, a specific RNA methylation called m5C has been identified. This modification is catalyzed by methyltransferases (writers), including NSUN1-NSUN6, TRDMT1, DNMT1, DNMT3A, and DNMT3B; dimethyltransferases (erasers), such as TET1, TET2, and TET3; and readers, such as ALYREF and YBX1. These proteins play crucial roles in the translation and degradation processes of downstream RNAs^1–4^. DNA methyltransferases (DNMTs) transfer the methyl group from S-adenosylmethionine (SAM) to the 5-carbon position of cytosine to produce 5-methylcytosine. The 5-carbon position of cytosine in RNA may also be modified by RNA methyltransferases by the addition of a methyl group. It is important to note that there are other enzymes called "erasers" and "readers" that can specifically identify and bind to m5C. Collectively, these three classes of enzymes regulate the levels and behavior of m5C in DNA and RNA^5^. The alteration of RNA m5C has emerged as a primary focus in cancer research at the epigenetic level. Various types of m5C readers play significant roles in cancer development by influencing processes such as cell division, migration, invasion, and drug sensitivity^6^.

The Aly/REF nuclear export factor (ALYREF), also referred to as THOC4, is an mRNA export adaptor that is a component of the transcription export complex (TREX)^7^. The most significant nuclear export factor, ALYREF, is mostly located around the 5' end of mRNA in vivo in a manner that is reliant on CBP80. On the other hand, ALYREF directly binds to PABPN1 and CstF64, which are recruited to mRNA nuclear output and 3′ processing and are related to the prognosis of HCC patients. Chen Xue reported that ALYREF mediates RNA m5C modification to promote hepatocellular carcinoma progression^8–10^. YBX1 was reported to be overexpressed in a number of cancers, including liver cancer, breast cancer, and bladder urothelial cancer, and it may be a clinical biomarker for prognosis^4,8,11,12^. The RNA methyltransferase NSUN2 catalyzes 5-methylcytosine, which was specifically identified by ALYREF to control RNA metabolism, particularly mRNA export^13^. ALYREF interacts with the NEAT1 promoter region in BRCA to increase the overall transcriptional activity of NEAT1 by stabilizing CPSF6^12^. Wang et al. showed that, in addition to indirectly activating PKM2 transcription, hypoxia-inducible factor-1α (HIF-1α) also activated ALYREF, which then bound to PKM2 m5C sites in 3'-untranslated regions, stabilized PKM2, and encouraged the growth of bladder cancer cells through PKM2-mediated glycolysis^14^. ALYREF improved the exosome secretion effect and increased the stability of YAP mRNA, which is beneficial for future LUAD treatment. YAP m5C alteration takes place in the 328–331 3' UTR where it is found^15^.

YBX1 functions as an RNA-binding protein and a transcription factor in the nucleus and cytoplasm, facilitating DNA transcription, replication, chromatin remodeling, pre-mRNA splicing, and translation initiation through its variable N-terminal domain rich in alanine and proline (A/P domain)^16,17^. According to reports, YBX1 is overexpressed in several carcinomas, such as bladder, breast, renal cell, and prostate, and it has been hypothesized that it serves as a clinical biomarker with a poor prognosis^18–23^. Recent studies have reported that YBX1, as a m5C binding protein, plays a role in facilitating mRNA export and stabilization in bladder cancer by recognizing m5C. Specifically, in urinary bladder cancer （UBC）, YBX1 recognizes m5C-modified mRNAs through the indole ring of W65 and maintains the stability of ELAVL1, which was revealed by single-nucleotide resolution landscape analysis of messenger RNA m5C modifications and rigorous experimental investigations^13,18,24^. Through an mRNA 3'-UTR m5C-methylation site in HBGF, YBX1 and NSUN2 work together to promote m5C-regulated oncogene activation in UBC^18^. Wang L, Zhang J, and others proved that YBX1, as a reader protein involved in NSUN2, mediated the stabilization of KRT13 and enhanced the level of KRT13. In cervical cancer, the NSUN2 m5C-KRT13-YBX1 oncogenic regulatory pathway encourages cell migration and invasion^25^.

Although ALYREF and YBX1 have been identified in the previously mentioned cancer types, m5C readers have not been thoroughly investigated in the context of the complete cancer lineage. To enhance the understanding of m5C readers in cancers, we utilized publicly available databases, specifically The Cancer Genome Atlas (TCGA) and Genotype-Tissue Expression (GTEx) databases. These resources provide valuable insights into the molecular mechanisms and clinical diagnosis of ALYREF and YBX1, thus contributing to a more comprehensive understanding of m5C readers in cancer biology. We carried out a systematic analysis of the expression and prognosis in 33 different cancer types to understand the potential roles of m5C readers (ALYREF and YBX1) and investigated the association between the expression of ALYREF, YBX1 and immune-related genes in several cancers (including KIRP, LGG and LIHC). By thoroughly analyzing ALYREF and YBX1, we aim to provide a solid basis for the identification of novel biomarkers for the early diagnosis of cancer and the prognosis of therapy.

## Results

### Expression and prognostic value of ALYREF and YBX1 in different types of cancers

**Figure 1:**
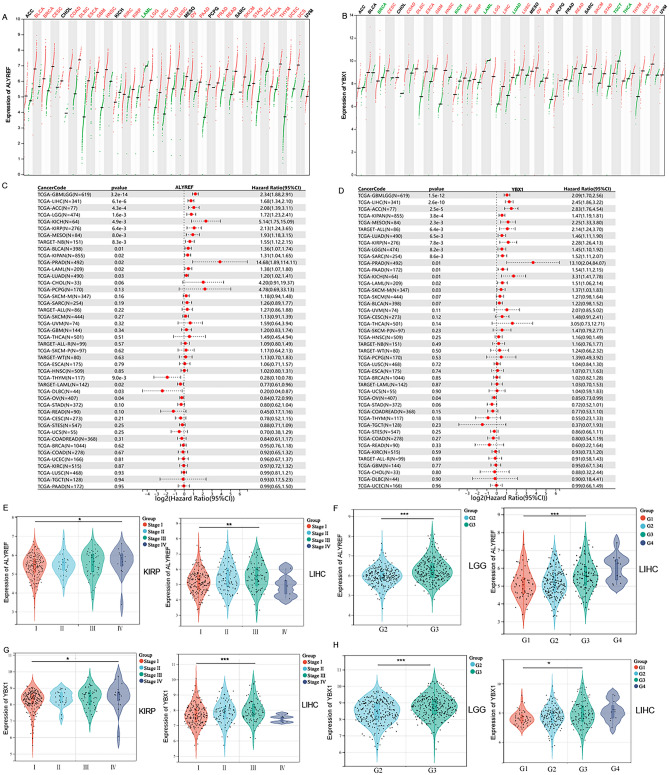
Expression and prognostic value of ALYREF and YBX1 in different types of cancers. (**A**,**B**) The expression levels of (**A**) ALYREF and (**B**) YBX1 were assessed across 33 cancer types using data obtained from the TCGA and GTEx datasets, with red indicating upregulation and green indicating lower expression. (**C**,**D**) Hazard ratio of (**C**) ALYREF and (**D**) YBX1 gene expression with OS, by the Cox regression analysis method in different types of cancers. (**E**–**H**) The violin diagrams showing the correlation between ALYREF (**E**–**F**), YBX1 (**G**–**H**) and Tumor Node Metastasis, pathologic stage. *p*-Value < 0.05 is regarded as significant. (*p < 0.05, **p < 0.01 and ***p < 0.001).

The overall workflow of this pan-cancer analysis of m5C readers was demonstrated in Supplementary Fig. S4. Initially, the expression levels of ALYREF and YBX1 were collected in 33 different types of cancers from the TCGA and GTEx databases. The analysis revealed a statistically significant upregulation of ALYREF in 25 common cancers (Fig. 1A), including BLCA, BRCA, CESC, COAD, DLBC, ESCA, GBM, HNSC, KIRC, KIRP, LGG, LIHC, LUAD, LUSC, OV, PAAD, PRAD, READ, SKCM, STAD, TGCT, THCA, THYM, UCEC, and UCS. Conversely, ALYREF was found to be underexpressed only in LAML. Additionally, YBX1 showed statistically significant overexpression in 19 common cancers (Fig. 1B), including CESC, COAD, DLBC, ESCA, GBM, HNSC, KIRC, KIRP, LGG, LIHC, LUSC, OV, PAAD, READ, SKCM, STAD, THYM, UCEC, UCS, and was downregulated in 6 cancers, including BRCA, KICH, LAML, LUAD, TGCT, and THCA.

We divided the clinical database into two groups based on the expression levels of ALYREF and YBX1 to explore their association with the prognosis of pan cancers. The forest plot analysis of overall survival (OS) revealed a significant correlation between the expression of ALYREF, YBX1 and prognostic value across various cancers. Among the 10 tumor types (ACC, BLCA, KICH, KIRP, LGG, LIHC, LUAD, MESO, PAAD and PRAD), high expression of ALYREF was associated with poor prognosis, whereas the 3 tumor types (DLBC, THYM, OV) with low expression of ALYREF exhibited a similar unfavorable prognosis (Fig. 1C). In 9 tumor types (ACC, KIRP, LGG, LIHC, LUAD, MESO, PRAD, PAAD and SARC), high expression of YBX1 corresponded to an unfavorable prognosis, while one tumor type (OV) with low expression of YBX1 exhibited a poor prognosis (Fig. 1D). Moreover, the time-dependent ROC curve indicated that the 1-year, 3-year and 5-year OS of ALYREF were all above 0.5 in ACC, KIRP, LGG, LIHC and LUAD; at the same time, the 1-year, 3-year and 5-year OS of YBX1 were above 0.5 in KIRP, LGG, LIHC, PAAD and SARC (Supplementary Fig. S1).

Above all, ALYREF and YBX1 overexpression in KIRP, LGG, and LIHC was associated with shorter OS. Taking into consideration the aberrant expression and prognostic value of ALYREF and YBX1 across cancers, we selected KIRP, LGG and LIHC as the main cancer types for further investigation. A higher tumor stage signifies an increased level of tumor progression, while an elevated clinical grading level indicates a lower degree of cell differentiation. In these tumor types, high expression of ALYREF and YBX1 is observed in advanced pathologic TNM stages as well as clinical grade. Figure 1E,F shows that high expression of ALYREF was associated with elevated clinical stage (stage I vs. stage IV in KIRP, stage I vs. stage III in LIHC) and tumor grade (G2 vs. G3 in LGG, and G1 vs. G3 in LIHC). Similar results for YBX1 were shown in Fig. 1G, H. Collectively, these findings suggest that ALYREF and YBX1 hold promise as prognostic biomarkers for KIRP, LGG and LIHC.

### Correlation between the tumor microenvironment and the expression of ALYREF and YBX1

**Figure 2:**
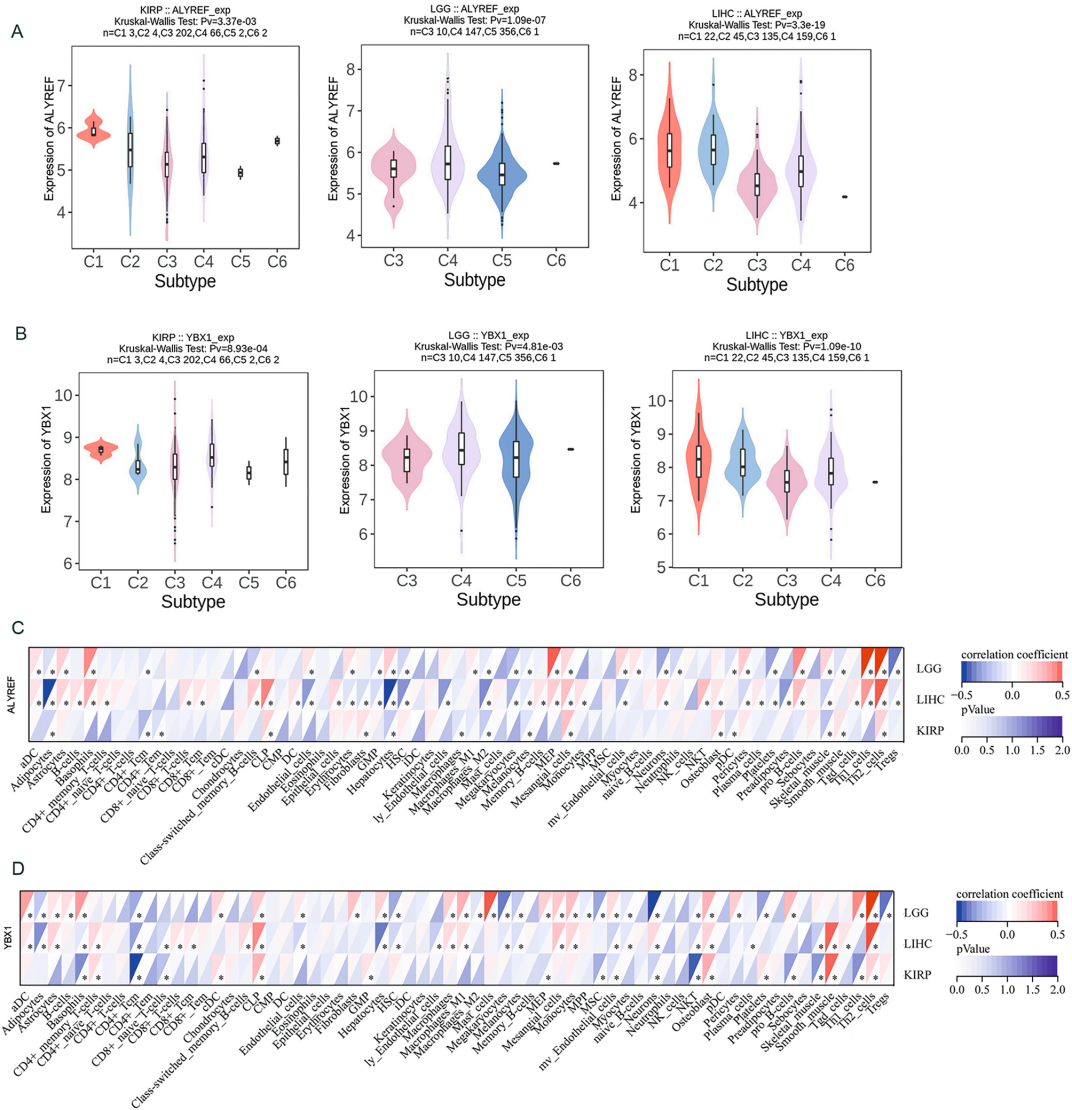
Correlation between the tumor microenvironment and expression of ALYREF, YBX1. (**A**,**B**) The violin diagrams showing the associations between the expression of (**A**) ALYREF, (**B**) YBX1 and immune subtypes across KIRP, LGG and LIHC. (**C**,**D**) The correlation of (**C**) ALYREF, (**D**) YBX1 expression levels and key immune cells (aDC, iDC, B cells, macrophages, neutrophil, dendritic cells and etc.) in KIRP, LGG and LIHC. (*p < 0.05, **p < 0.01 and ***p < 0.001).

We utilized TISIDB to analyze the expression of ALYREF and YBX1 in different immune subtypes, including the C1 (wound healing), C2 (IFN-blocking dominant), C3 (inflammatory), C4 (lymphocyte exhaustion), C5 (immune quiescent), and C6 (TGF-multinucleated dominant) subtypes. Figure 2A,B demonstrates the differential expression of ALYREF and YBX1 across these immune subtypes. Additionally, in KIRP, patients in C4 had a worse prognosis than those in C2. In LGG, patients in C4 had a worse prognosis than those in C5. In LIHC, patients in both C1 and C4 had a worse prognosis than those in C3 (Supplementary Fig. S2). We found that ALYREF and YBX1 were differentially expressed in different immune subtypes and that their prognostic value also varied across different immune subtypes. Immune subtypes are characterized by distinct compositions of the tumor microenvironment (TME), which may partially explain the varying roles of m5C readers in the prognosis of different cancers.

The TME is a complex network comprising diverse cellular and noncellular components, including immune cells, endothelial cells, fibroblasts, and various biomolecules. Targeting the TME has emerged as a promising therapeutic strategy for cancer treatment due to its crucial involvement in regulating tumor progression and influencing the response to standard-of-care therapies. To understand the interactions between the expression of m5C readers and the tumor microenvironment, we analyzed the correlations between expression of ALYREF and YBX1 and various immune cell populations. Our findings revealed a positive correlation between ALYREF and YBX1 expression in Th1/Th2 cells, B cells, basophils, and dendritic cells. Conversely, ALYREF and YBX1 expression was negatively correlated with regulatory T cells, CD4+ T cells, CD8+ T cells, NK cells and M2 macrophages (Fig. 2C,D).

### Potential association between immune-related factors and the expression of ALYREF and YBX1

Our analysis revealed a significant association between the expression of ALYREF and YBX1 and immune modulators, including chemokines, receptors, major histocompatibility complex (MHC) molecules, immunoinhibitory factors, and immunostimulatory factors. To further investigate the relationships between immune modulators and the expression of ALYREF and YBX1, we performed a coexpression analysis across the three tumors.

**Figure 3:**
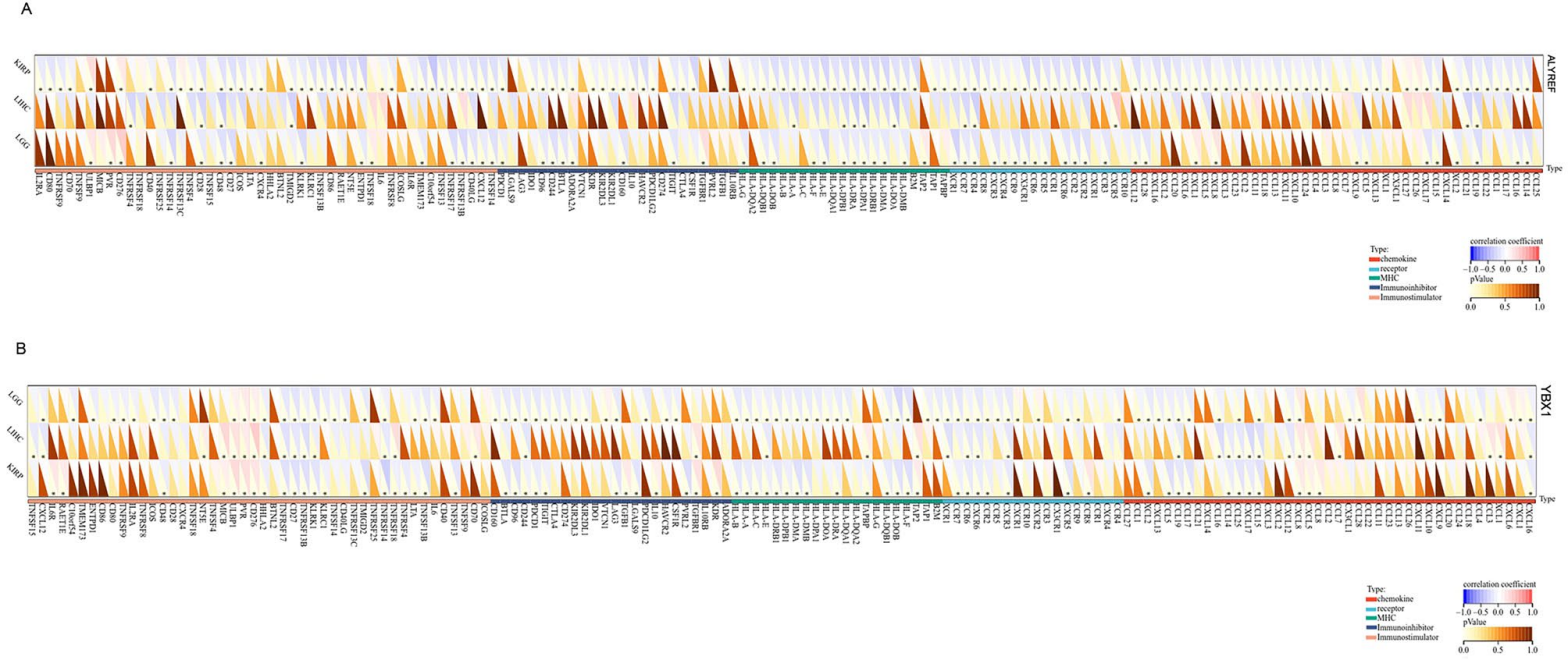
Potential association between immune-related factors and expression of ALYREF, YBX1. (**A**,**B**) Heatmaps showing the relationship between (**A**) ALYREF, (**B**) YBX1 expression and chemokines, receptors, MHC molecules, immunoinhibitory factors, and immunostimulatory factors across different cancer types. (*p <0.05).

Consistent with our results (Fig. 3A,B), we observed a strong correlation between ALYREF, YBX1 and most immune-related genes. Specifically, chemokines such as CCL19 and CCL21 exhibited negative correlations with ALYREF expression, and chemokines such as CCL5 and CXCL8 exhibited positive correlations with YBX1 expression. Moreover, immunostimulatory factors and immunosuppressive factors were tightly correlated with ALYREF and YBX1 expression. All these results demonstrated that high expression of ALYREF and YBX1 is significantly relevant to tumor immunity.

**Figure 4:**
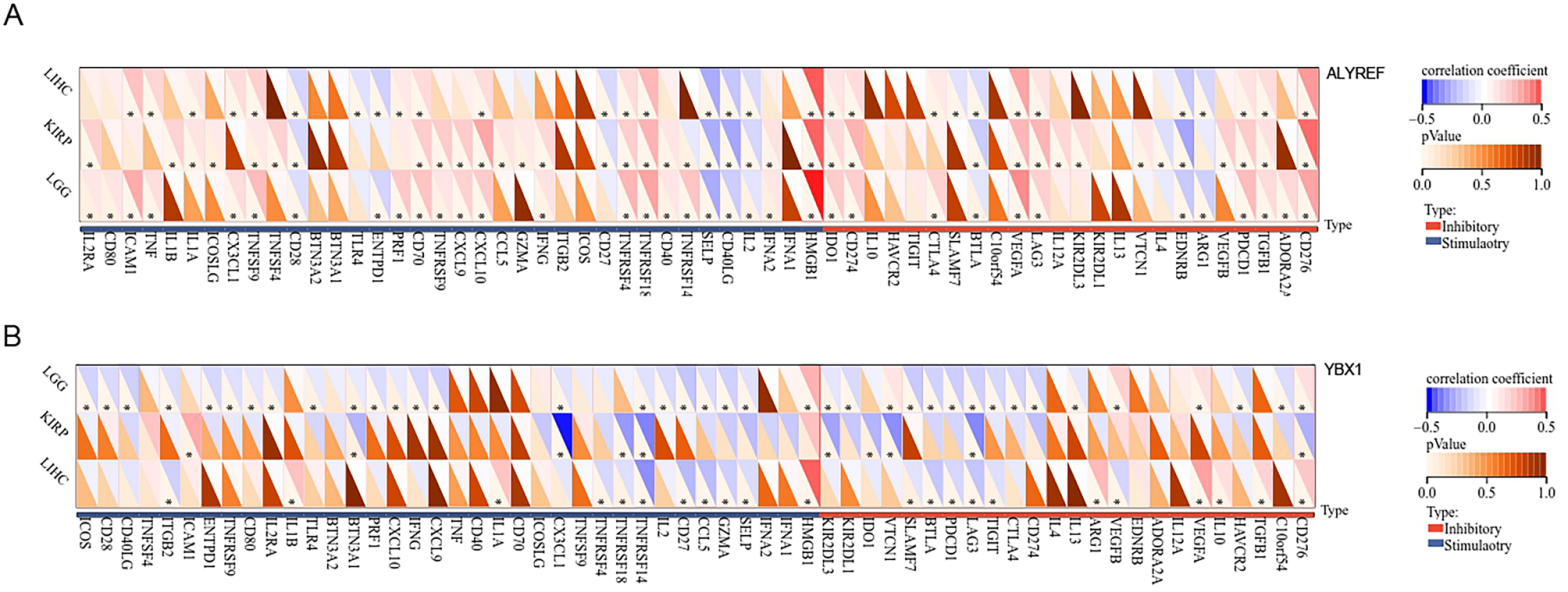
Potential association between checkpoints and expression of ALYREF, YBX1. (**A**,**B**) Heatmaps illustrating the association between (**A**) ALYREF, (**B**) YBX1 expression and inhibitory checkpoints and stimulatory checkpoints. (*p < 0.05).

Immune checkpoints refer to programmed death receptors and their ligands^26^. Numerous illnesses, including cancer, may be caused by aberrant immune checkpoint expression^27,28^. Immune checkpoints are triggered by chemicals released by tumor cells, which allows them to persist by suppressing T-cell immune activity^28^. Previous studies have demonstrated the significant influence of immune checkpoint genes on immune cell infiltration and immunotherapy^29^. Therefore, we investigated the associations between the expression of ALYREF, YBX1 and immune checkpoint protein (ICP) genes in KIRP, LGG and LIHC. Our analysis revealed a strong correlation between ALYREF expression and more than 60% of the ICP genes, particularly CD276, PDCD1, CD40LG and SELP (Fig. 4A). Similarly, our analysis demonstrated a significant correlation between YBX1 expression and over 50% of the ICP genes, with notable associations observed for CD276, CX3CL1 and ITGB2 (Fig. 4B). These findings suggest that ALYREF and YBX1 proteins have the potential to serve as targets for immunotherapy, enabling the prediction of immunotherapy response and leading to promising therapeutic outcomes.

### TIDE scores and the expression of ALYREF and YBX1

**Figure 5:**
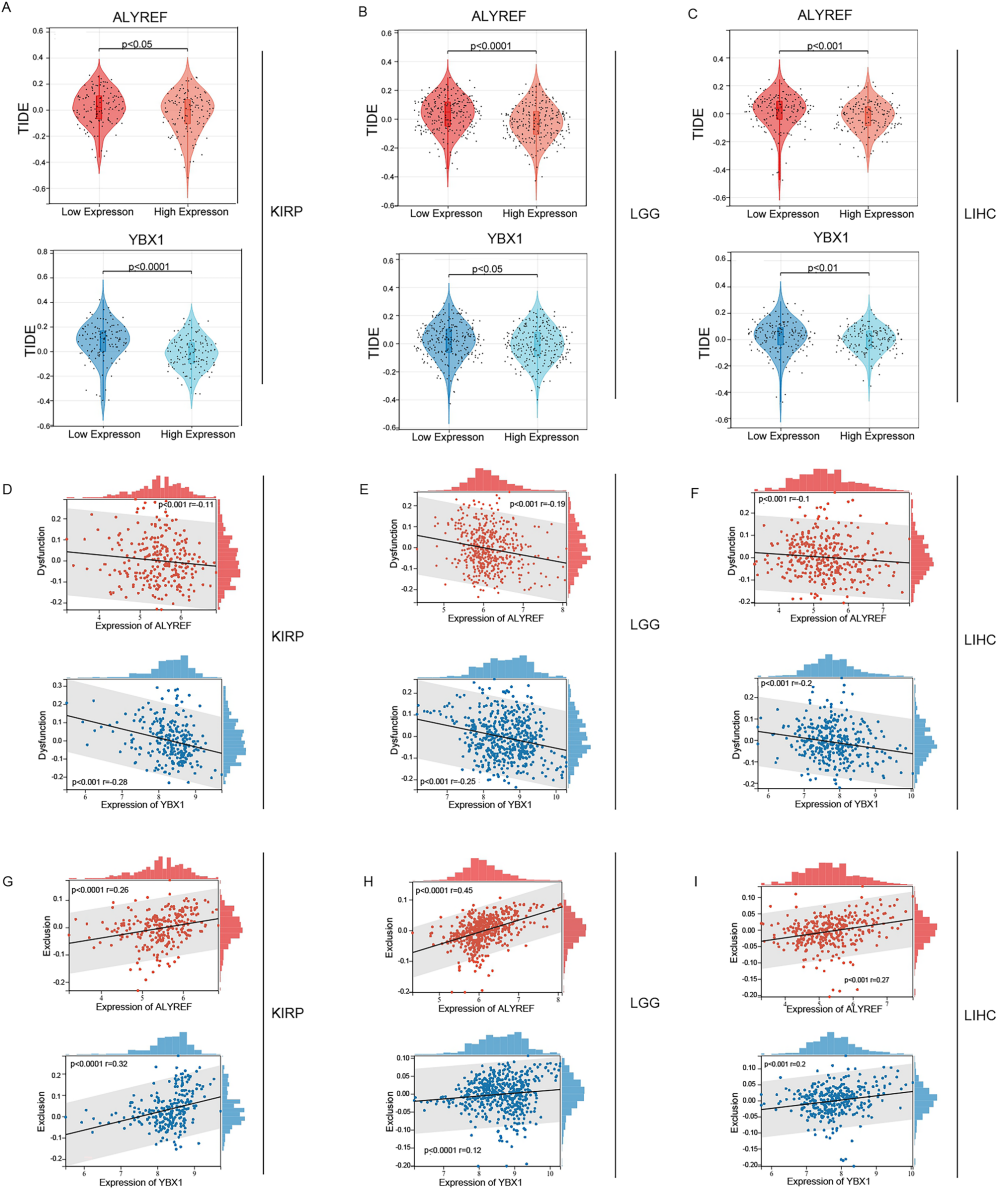
TIDE scores and the expression of ALYREF, YBX1. (**A**–**C**) Violin plot shows the TIDE scores for high and low expression groups of ALYREF and YBX1. From left to right are KIRP (**A**), LGG (**B**) and LIHC (**C**). (**D**-**F**) Correlation analysis of Dysfunction and expression of ALYREF and YBX1 in KIRP (**D**), LGG (**E**) and LIHC (**F**). (**G**-**I**) Correlation analysis of Exclusion and expression of ALYREF and YBX1 in KIRP (**G**), LGG (**H**) and LIHC (**I**). (*p < 0.05, **p < 0.01, ***p < 0.001, ****p < 0.0001).

We use the TIDE algorithm to predict the ICB response. It is well known that higher TIDE scores are associated with poorer immunotherapy^30^. Figure 5A–C shows that the higher expression group of ALYREF and YBX1 in KIRP, LGG and LIHC had a lower TIDE score, which suggested that patients with high expression of ALYREF and YBX1 may be more sensitive to ICB therapy. T-cell dysfunction was more significant in the low expression of ALYREF and YBX1. However, immune exclusion was observed more frequently in the high expression of ALYREF and YBX1 (Fig. 5D–I).

### Analysis of drug sensitivity and resistance in ALYREF and YBX1

**Figure 6:**
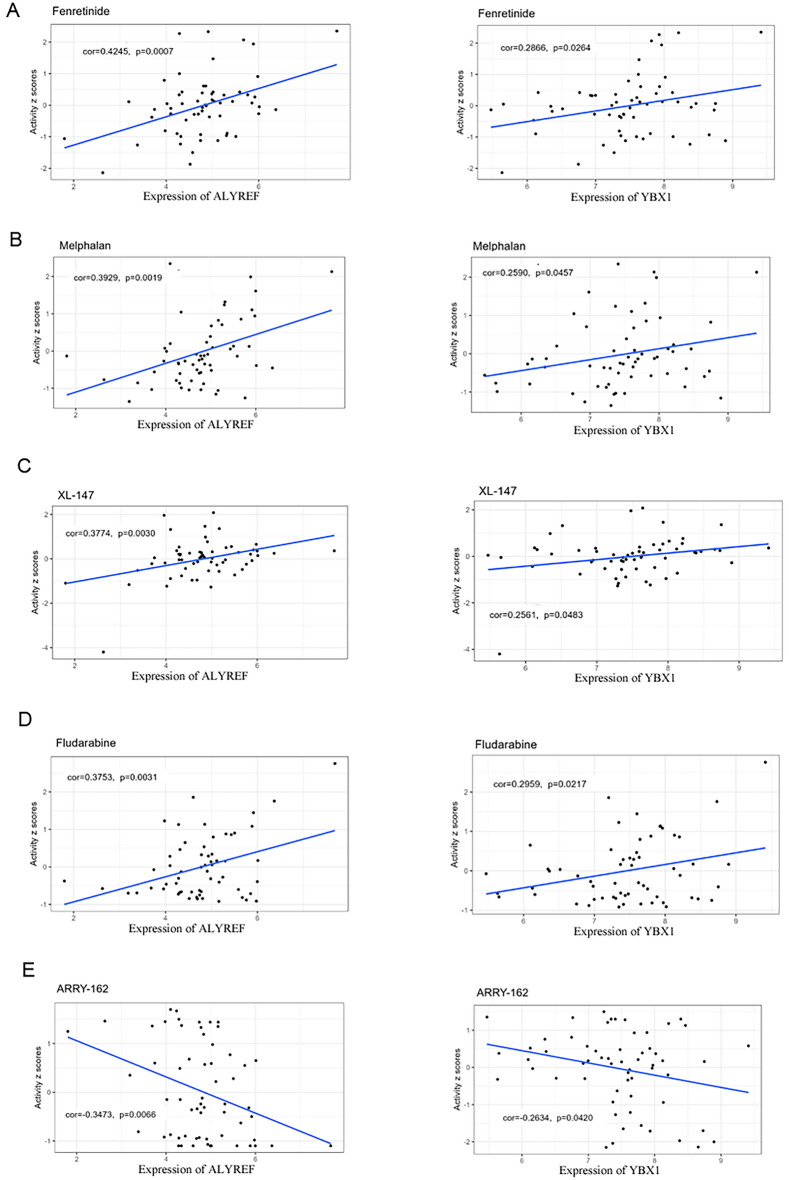
Drug sensitivity and resistance analysis of ALYREF and YBX1. The expressions of ALYREF and YBX1 were associated with the sensitivity of (**A**) Fenretinide (**B**) Melphalan (**C**) XL-147 (**D**) Fludarabine, (**E**) ARRY-162. X axis is the gene expression, and Y axis is the drug activity z scores values. The left panel represents ALYREF, while the right panel represents YBX1.

Drug sensitivity and resistance are crucial for cancer therapy, especially resistance, which develops toward conventional therapy and is one of the important reasons for chemotherapy failure in cancer^31^. To investigate potential correlations between drug sensitivity/resistance and the expression levels of ALYREF and YBX1, we analyzed data from the CellMiner database. Our results revealed a significant positive correlation between the expression of ALYREF and YBX1 and drugs (fenretinide, melphalan, XL-147, and fludarabine) (Fig. 6A–D). Figure 6E shows the negative correlation between the expression of ALYREF and YBX1 and the drug ARRY162.

### Positively pathway enrichment analyses for ALYREF and YBX1

**Figure 7:**
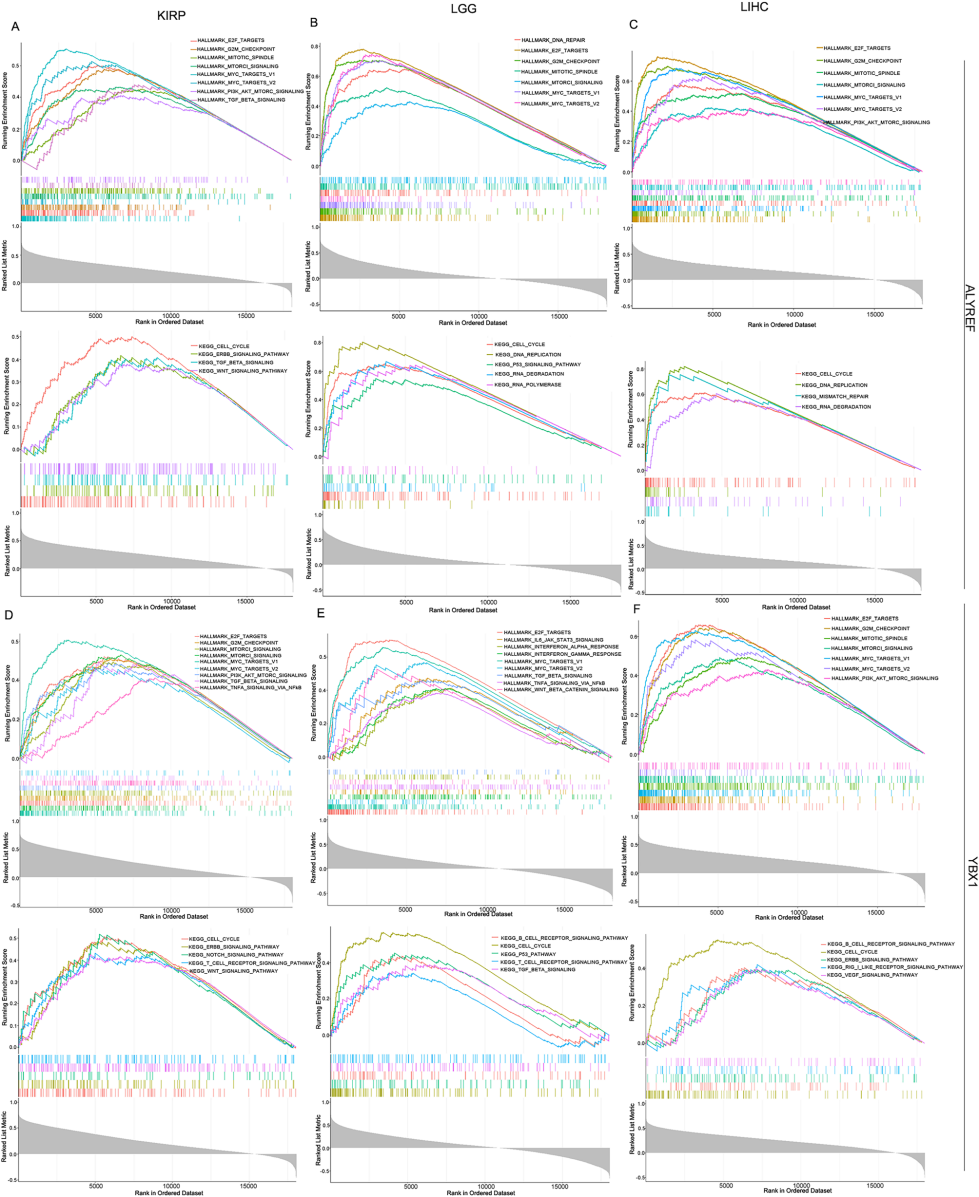
Positively pathway enrichment analyses for ALYREF and YBX1. Pathway enrichment analyses were performed for (**A**–**C**) ALYREF and (**D**–**F**) YBX1. The upper panel represents hallmark pathways, while the bottom panel represents KEGG pathways. (*p < 0.05, FDR < 0.5).

To assess the functional enrichment of the expression of ALYREF and YBX1, we performed single-sample gene set enrichment analysis. As depicted in Fig. 7A–F, the HALLMARK and KEGG^32–34^ pathway analyses revealed a positive association between the expression of ALYREF and YBX1 and various fundamental processes in KIRP, LGG and LIHC. These processes included DNA replication, E2F, Myc targets, mTORC1, and the cell cycle. Notably, immune-related signaling pathways and essential signal transduction pathways related to immune responses were significantly enriched, such as the PI3K/AKT/mTOR pathways, T-cell receptor, IL6/JAK/STAT3, B-cell receptor, WNT/β-catenin pathway, INFα, TNFα, JAK/STAT and TGF pathways.

Taken together, these findings suggest that ALYREF and YBX1, as essential m5C readers, may promote the transcription and translation of target genes through coexpression with other genes, thereby influencing the immune response in KIRP, LGG and LIHC.

### Functional enrichment analysis of ALYREF, YBX1 and coexpressed genes

**Figure 8:**
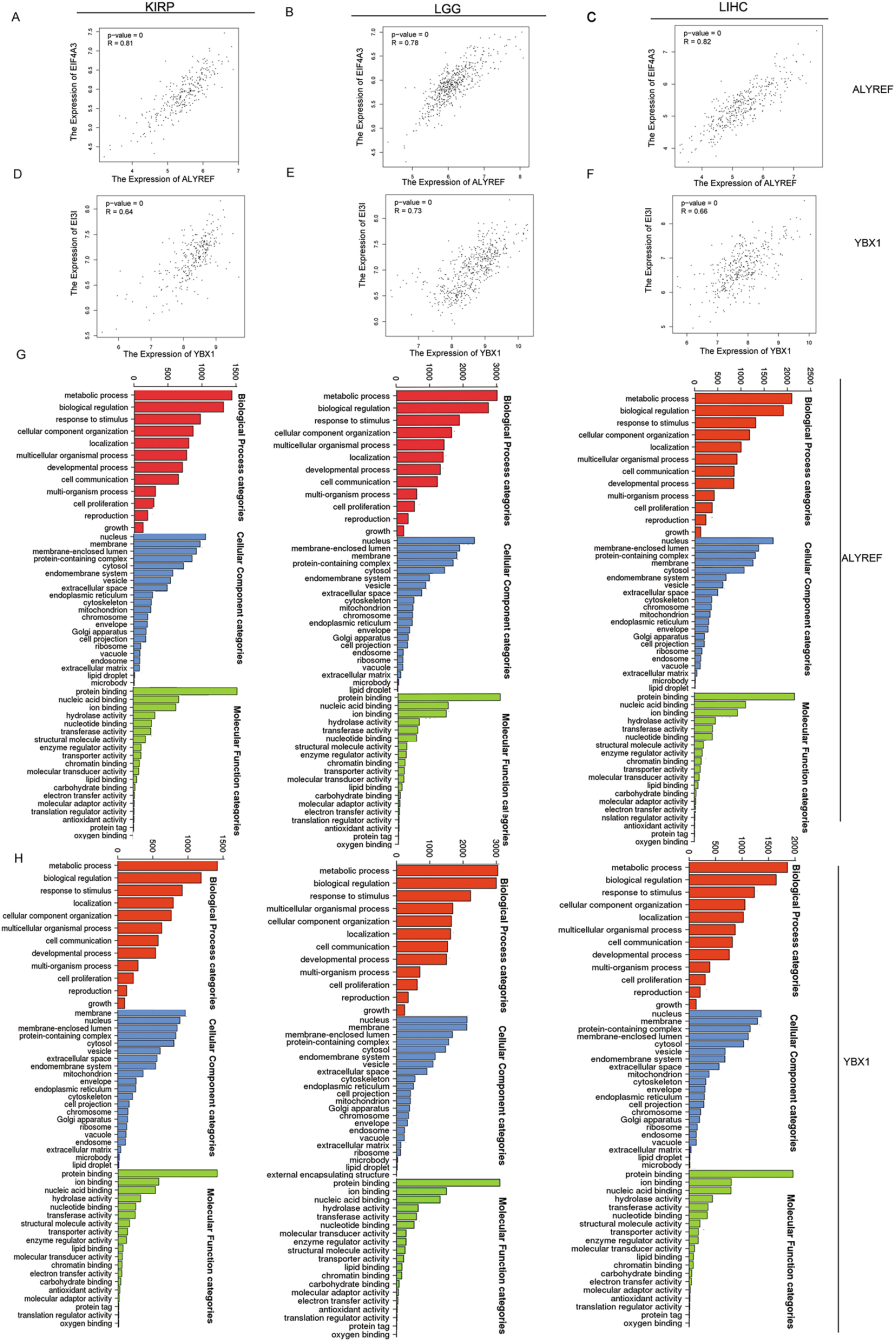
Functional enrichment analysis of ALYREF, YBX1 and Co-expressed genes. (**A**–**C**) The correlation with ALYREF and eIF4A3 in KIRP, LGG and LIHC. (**D**–**F**) The correlation with YBX1 and eIF3I in KIRP, LGG and LIHC. (**G**–**H**) The GO analysis indicated that (**G**) ALYREF, (**H**) YBX1 and eIF family have many co-related pathways in common.

Using LinkedOmics, we identified the top 50 positively associated differentially expressed genes (DEGs) for ALYREF and YBX1. Supplementary Fig. S3A–F reveals a close relationship between ALYREF/YBX1 and the eIF family, which includes EIF4A3, EIF6, EIF3I, and EIF3H. Using GEPIA analysis, we observed that ALYREF expression was strongly correlated with eIF4A3 expression and that YBX1 expression was strongly correlated with eIF3I (Fig. 8A–F). Subsequently, we identified positively associated DEGs for ALYREF, YBX1 and eIFs. As shown in Fig. 8G–H, for common DEGs, GO and KEGG analyses showed that metabolic processes were significantly enriched in the biological processes category, and nuclei were significantly enriched in the cellular component category. For the molecular function component, protein binding was significantly enriched.

## Discussion

The m5C represents a widespread and important DNA/RNA modification that has been implicated in various diseases^1,13^. ALYREF and YBX1 have been identified as crucial m5C reader proteins that specifically bind to modified sites to recognize m5C-containing oligonucleotides^6^. Their role in the recognition of m5C modifications is essential for the proper functioning and regulation of various biological processes^6^. ALYREF and YBX1 are both implicated in numerous RNA processing events, and their abnormal expression has been linked to reduced survival rates in cancer patients^1,4,7,18,35–39^. However, there is a lack of comprehensive research discussing the overall landscape and mechanism of m5C reader proteins across different cancers. This study provides a comprehensive analysis of ALYREF and YBX1 in pancancer, encompassing expression profiles, prognostic implications, correlated genes, immune infiltration, and potential pathways.

We discovered noteworthy changes in the expression of ALYREF and YBX1 among 33 tumor types compared to corresponding normal tissues. Specifically, these genes were upregulated in 19 prevalent cancers (including CESC, COAD, DLBC, ESCA, GBM, HNSC, KIRC, KIRP, LGG, LIHC, LUSC, OV, PAAD, READ, SKCM, STAD, THYM, UCEC, UCS) and downregulated in LAML. Furthermore, the expression levels of these genes were significantly correlated with the clinical characteristics of cancer patients.

Recent studies have demonstrated a significant correlation between m5C modification and cancer progression^3,40^. Previous research has examined six categories of immune infiltration (C1–C6) in cancer patients, potentially influencing tumor cell growth^41^. The C1 subtype, associated with wound healing, and the C2 subtype, characterized by IFN-blocking dominance, are considered poor cytotoxic immunophenotypes. These subtypes exhibit enrichment in angiogenic gene expression, M1/M2 macrophage polarization, CD8+ T-cell signaling, and TCR diversity^42–44^. The C3 subtype, characterized by inflammation, and the C4 subtype, associated with lymphocyte exhaustion, exhibit intermediate cytotoxicity with a significant contribution from the immunosuppressive component. These subtypes show enrichment in invasion-related processes such as epithelial-to-mesenchymal transition, focal adhesion, and extracellular matrix remodeling^29,45–47^. The C5 subtype, known as immune quiescent, and the C6 subtype, characterized by TGF-multinucleated dominance, exhibit high cytotoxicity. These subtypes are associated with the upregulation of multiple metabolic pathways involved in oxygen free radical production, as well as the upregulation of the antigen presentation machinery and IFN signaling^48^.

The TME comprises a complex network of diverse cellular and noncellular elements, such as immune cells, endothelial cells, fibroblasts, and other biomolecules. Interactions between cancer cells and different TME components promote immune evasion and ultimately drive the enhanced proliferation and invasiveness of cancer cells. These processes are closely associated with tumor recurrence and patient survival^49,50^.

On the one hand, our findings confirmed that there was a negative correlation between the expression of m5C readers (including ALYREF and YBX1) and CD4+ T cell, CD8+ T cell, NK cell, and regulatory T-cell (Treg cell) infiltration. CD4+ T-cell and CD8+ T-cell tumor infiltration is one of the key characteristics of effective cancer immunotherapy. and changes the TME to promote antitumor immunity^51,52^. Possessing multiple cytotoxicity mechanisms and the ability to modulate the immune response through cytokine production, NK cells play a pivotal role in anticancer immunity^53^. Treg cells have the ability to limit the function of antigen-presenting cells by CTLA-4-dependent downregulation of CD80 and CD86, thereby playing a detrimental role in suppressing cancer progression by evading immune surveillance and suppressing the antitumor immune response^54,55^. On the other hand, the expression levels of ALYREF and YBX1 were positively correlated with the infiltration levels of T helper 2 cells (Th2 cells), which contributed to the formation of an immunosuppressive TME. Following differentiation, Th2 cells secrete IL-4, IL-5, IL-10, IL-13, and IL-17 and eventually undergo tumor growth and metastasis^56,57^. At the same time, we also found that the expression of ALYREF and YBX1 was highly associated with chemokines, chemokine receptors, and MHC genes in KIRP, LGG and LIHC. These results implied that ALYREF and YBX1 might exert a significant role in the immune response of tumor cells to immunotherapy.

Overexpression of ALYREF and YBX1 was accompanied by the upregulation of immune checkpoint inhibitors (such as CD276 and PDCD1) and downregulation of immune checkpoint stimulants (such as CX3L1, ITGB2, CD40LG and SELP), which promoted evasion of immune surveillance by these tumor cells. In summary, these results provide supportive evidence that ALYREF and YBX1 are linked to the immunosuppressive microenvironment in cancers.

We analyzed the relationship between TIDE scores and the expression of ALYREF and YBX1 to further test the response to immunotherapy and found that the high-expression group of ALYREF and YBX1 had a lower TIDE score, indicating that the high-expression of m5C readers (ALYREF and YBX1) may respond better to immunotherapy. These results suggest that m5C readers (ALYREF and YBX1) could predict the effectiveness of immunotherapy in patients with KIRP, LGG and LIHC.

Furthermore, in the drug sensitivity and resistance analysis, we found that the expression of ALYREF and YBX1 was positively correlated with resistance to chemotherapy (fenretinide, melphalan, XL-147, and fludarabine) and positively correlated with sensitivity to chemotherapy (ARRY162) in various cancers, which indeed provides valuable insights for future research on increasing chemotherapy sensitivity and combating chemotherapy resistance. Data presented by Tao et al. showed the same results that multidrug resistance can be reversed by targeting the YBX1 signaling cascade^58,59^.

Last, we observed a significant correlation between expression (ALYREF and YBX1) and members of the eIF family, including eIF4A3, eIF3I, eIF3H, and eIF6. These proteins play crucial roles in preinitiation complex formation, mRNA translation initiation, and regulation of RNA metabolism through involvement in RNA splicing and other related processes^60,61^. Previous studies have reported significant upregulation of eIF3H and eIF3I in various malignancies^62^. Conversely, eIF3I activates Akt signaling by interacting with Clusterin, leading to increased expression of matrix metalloproteinase 13 (MMP13) and subsequent metastasis in HCC^63^. Additionally, multiple studies have demonstrated the overexpression of eIF4A3 in various malignancies, including HCC, gastric cancer, epithelial ovarian cancer, and ovarian cancer. Zhou et al. discovered that eIF4A3 binded to noncoding RNAs in cancer cells, thereby promoting cellular processes such as proliferation, migration, the Wnt/β-catenin signaling pathway, and epithelial-mesenchymal transition (EMT)^64–67^. Importantly, eIF4A3 has been demonstrated to facilitate the binding of ALYREF not only at spliced RNA sites but also at single-exon transcript sites^68^. The overactivation of eIF6 has been confirmed to be associated with tumor proliferation and invasion through the AKT and mTOR pathways in oral squamous cell carcinoma, glioblastoma, and colorectal cancer^69–74^. Our DEGs analysis revealed a significant correlation between m5C readers and the expression of eIFs in KIRP, LGG and LIHC. eIF4A3 can stimulate ALYREF binding at sites of spliced RNAs and single-exon transcripts. Interestingly, the m5C binding mode of YBX1 is similar to DNA m5C recognition by certain transcription factors^75,76^. Based on functional enrichment analysis, ALYREF, YBX1 and DEGs were mainly enriched in the DNA replication, E2F, Myc-targets, mTORC1, PI3K/AKT/mTOR pathways, T-cell receptor, IL6/JAK/STAT3, B-cell receptor, WNT/β-catenin pathway, INFα, TNFα, JAK/STAT and TGF pathways. Through meticulous functional annotation and rigorous enrichment analysis of differentially expressed genes, we successfully elucidated the impact of eIFs, specifically eIFs-m5C readers, on mRNA translation and RNA metabolism. These processes ultimately resulted in elevated levels of cellular m5C via the involvement of m5C readers (ALYREF and YBX1).

These findings suggest that ALYREF and YBX1, as m5C readers, have the potential to be biomarkers or novel targets for combined modality therapy and lead to favorable treatment outcomes. Until now, the m5C readers have mainly included ALYREF and YBX1, in the future there may be further exploration to discover more readers.

We acknowledge some limitations in our study. It primarily relies on data analysis using several online databases and lacks certain experimental evidences to verify our hypotheses. In future research, we will intend to address these limitations by investigating the precise mechanism between eIFs and m5C readers. In most cases of this study, we grouped the data into high-expression and low-expression groups using the median expression, which is an analysis shortage. Duo Yun constructed a score (m5C-score) based on the expression of m5C-related prognostic DEG expression to predict the prognosis of patients^77^, which is a worth learning idea using multivariate Cox analysis to avoid potential bias as much as possible. In the future, we may perform subgroup analysis by the expression of m5C readers and m5C scores to explore the potential of m5C readers in treatment.

## Conclusion

Our findings demonstrated that ALYREF and YBX1, as m5C readers, are aberrantly expressed in most cancers and are associated with disease prognosis. Additionally, they are correlated with the TME and drug sensitivity, particularly in KIRP, LGG and LIHC. Moreover, ALYREF and YBX1 were found to be positively associated with eIF family genes and enriched in various fundamental processes and immune responses. Therefore, targeting m5C readers offers an intriguing therapeutic strategy.

## Materials and methods

### Acquisition of gene expression data

The data for 10,496 TCGA (https://gdc.cancer.gov) samples in 33 different types of tumor tissues, as well as pertinent clinical information (type, survival status, clinical and pathological stage) integrated with the GTEx resource, were obtained from the UCSC Xena database (https://commonfund.nih.gov/GTEx/), and then log2(TPM + 1) transformation was performed^78–81^.

### Survival and prognostic analysis

The prognostic values of ALYREF and YBX1 in malignancies were assessed by a type of clinical outcome: OS. Prognostic indicators were assessed on criteria covering hazard ratios (HR), 95% confidence intervals and *p* values indicated when *p* < 0.05 was considered statistically significant. The time-dependent ROC curve was generated using the timeROC of R language to explore the prognostic value of ALYREF and YBX1.

### Immune subtype and tumor microenvironment

A tool for TCGA analysis was made available on the Sangerbox website (http://sangerbox.com) to estimate the stromal and immune cells in tumor samples. The potential relationships between the expression of ALYREF and YBX1 and the immune subtypes were discussed by using the TISIDB website (http://cis.hku.hk/TISIDB/) with the R package "IOBR" (which includes xCELL)^82^ in pancancer^83,84^.

### Factors related to immunity

We examined the link between ALYREF, YBX1 and more than 50 immune checkpoint-related genes in KIRP, LGG and LIHC on the Sangerbox website using the Pearson correlation test. Furthermore, the Sangerbox website was used to discuss the probable connection between the expression of m5C readers and immunomodulators (immune inhibitors, immune stimulators, MHC molecules and receptors, and chemokines).

### TIDE score analysis

TIDE score has been applied to predict responses to immune checkpoint blockade and determine mechanisms underlying tumor immune escape based on myeloid‐derived suppressor cells (MDSCs), M2 macrophages, and T‐cell dysfunction and exclusion^30,85^. The TCGA pan‐cancer TIDE scores, dysfunction scores and exclusion scores were directly downloaded from TIDE (http://tide.dfci.harvard.edu).

### Drug sensitivity and resistance analysis

To examine the drug sensitivity and resistance of ALYREF and YBX1 in pancancer, NCI-60 compound activity data and RNA-seq expression profiles were downloaded from CellMiner™ (https://discover.nci.nih.gov/cellminer/home.do)^86^. Drugs that have undergone FDA approval or clinical trials were chosen for investigation.

### Construction of ALYREF and YBX1 coexpression networks

We utilized the LinkedOmics database (http://www.linkedomics.org/)^87^ to identify DEGs associated3 with ALYREF and YBX1 and evaluated associated DEGs with the GEPIA2 web server (http://gepia2.cancer-pku.cn) using Spearman's correlation coefficients^88^. Using GO tools, we annotated pathway enrichments for positively common genes on DAVID (https://david.ncifcrf.gov/)^89,90^. The three domains of the GO analyses, molecular functions (MF), biological processes (BP), and cellular components (CC), were visualized using the bioinformatics website (http://www.bioinformatics. com.cn/).

### GSEA analysis

Single-sample gene set enrichment analysis (ssGSEA) denotes GSEA for a single sample, and both the procedure for calculating ES and sequencing the gene list rely on the gene expression levels in the sample. Using the "GSEA" R package, we investigated the probable biological functions of m5C readers based on a false discovery rate (FDR) < 0.05 and a *p* value < 0.05^91^.

### Statistical analyses

The data in this study are shown as the means with standard deviations (SD). The differences between the two groups were evaluated using t tests. R 4.1.3 was utilized to carry out statistical analyses. *p* < 0.05 (two-tailed) was regarded as statistically significant.

### Supplementary Information

Supplementary Figures.

## Data Availability

The datasets used and/or analyzed during the current study are available from the corresponding author upon reasonable request.
