# Supplementary material for: Multi-omic analyses of m5C readers reveal their characteristics and immunotherapeutic proficiency
Source: Sci Rep. 2024 Jan 18;14:1651. doi: 10.1038/s41598-024-52110-7 (PMC10796763; doi:10.1038/s41598-024-52110-7)
Supplement: Supplementary file 1 — Supplementary Figures. [file 41598_2024_52110_MOESM1_ESM.docx]

**Multi-Omic analyses of m5C Readers reveal their characteristics and immunotherapeutic proficiency**

Rui Xu^3^, Yue Wang^2^*, Ye Kuang^1^*

*^1^Department of Medical Laboratory, Yan'An Hospital of Kunming city, Kunming, Yunnan Province, China*

*^2^Department of Endocrinology, the First Affiliated Hospital of Dalian Medical University, Dalian,* *Liaoning Province, China.*

*^3^Department of Development Planning, International Medical Opening-up Pilot Zone (China), Fangchenggang, Guangxi Province, China*

ORCID of Corresponding Author:

Ye Kuang, ORCID: https://orcid.org/0000-0003-4946-3447

Yue Wang, ORCID: https://orcid.org/0000-0005-2475-1794

*Corresponding Author:

Ye Kuang

Address for Correspondence:

Department of Medical Laboratory,

Yan'An Hospital of Kunming city,

Kunming, Yunnan Province, China

Email address: [kyking4@126.com](mailto:kyking4@126.com)

Yue Wang

Address for Correspondence:

Department of Endocrinology,

the First Affiliated Hospital of Dalian Medical University,

Dalian, Liaoning Province, China

Email address: [wangyue@firsthosp-dmu.com](mailto:wangyue@firsthosp-dmu.com)


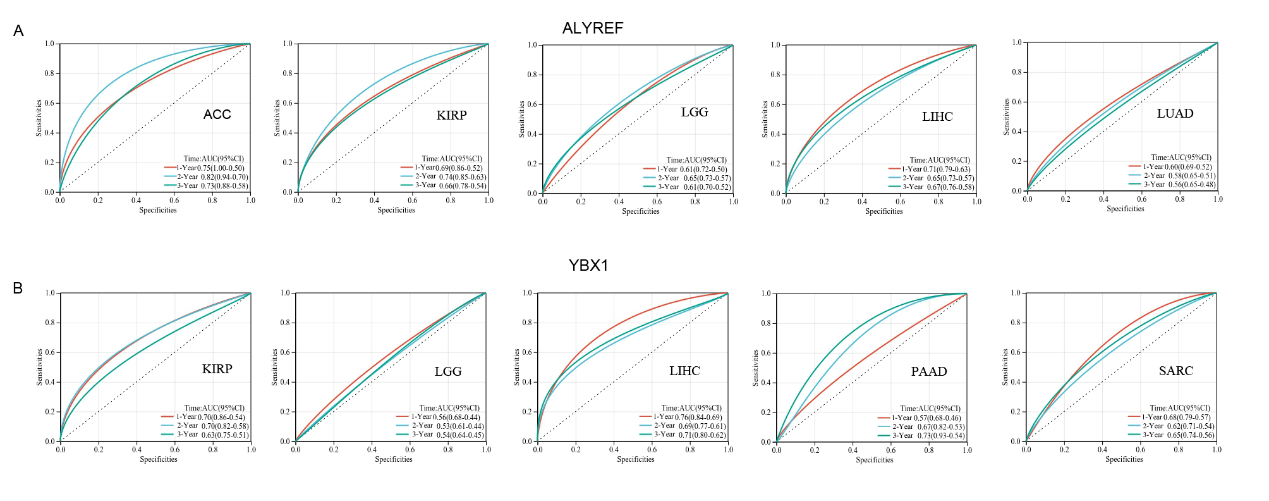


Figure S1. Prognostic value of ALYREF and YBX1. (A)For ALYREF, the time-dependent ROC curve of 1-year, 3-year and 5-year OS in ACC, KIRP, LGG, LIHC and LUAD. (B) For YBX1, the time-dependent ROC curve of 1-year, 3-year and 5-year OS in KIRP, LGG, LIHC, PAAD and SARC.


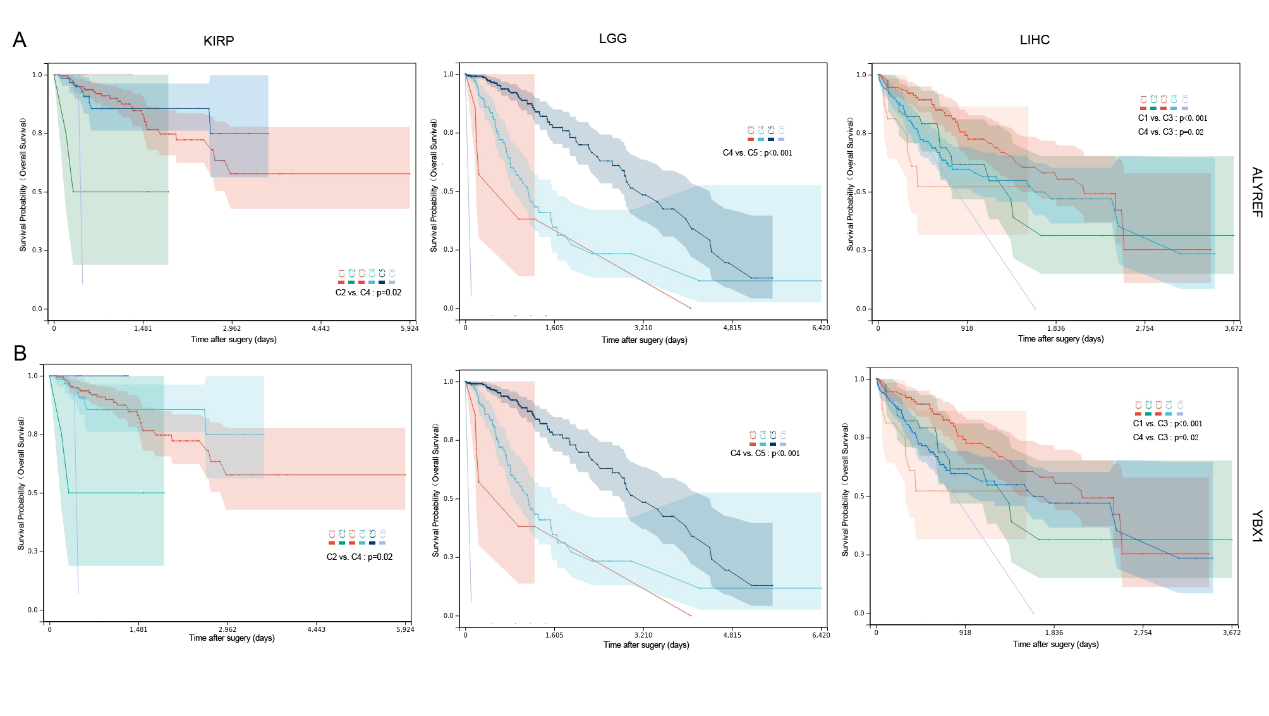


Figure S2. Kaplan–Meier analysis of different immune subtypes. Prognosis value analyses of C1-C6 immune subtypes for (A)ALYREF and (B)YBX1. From left to right, there represent KIRP, LGG and LIHC. The significance is determined by log-rank test.


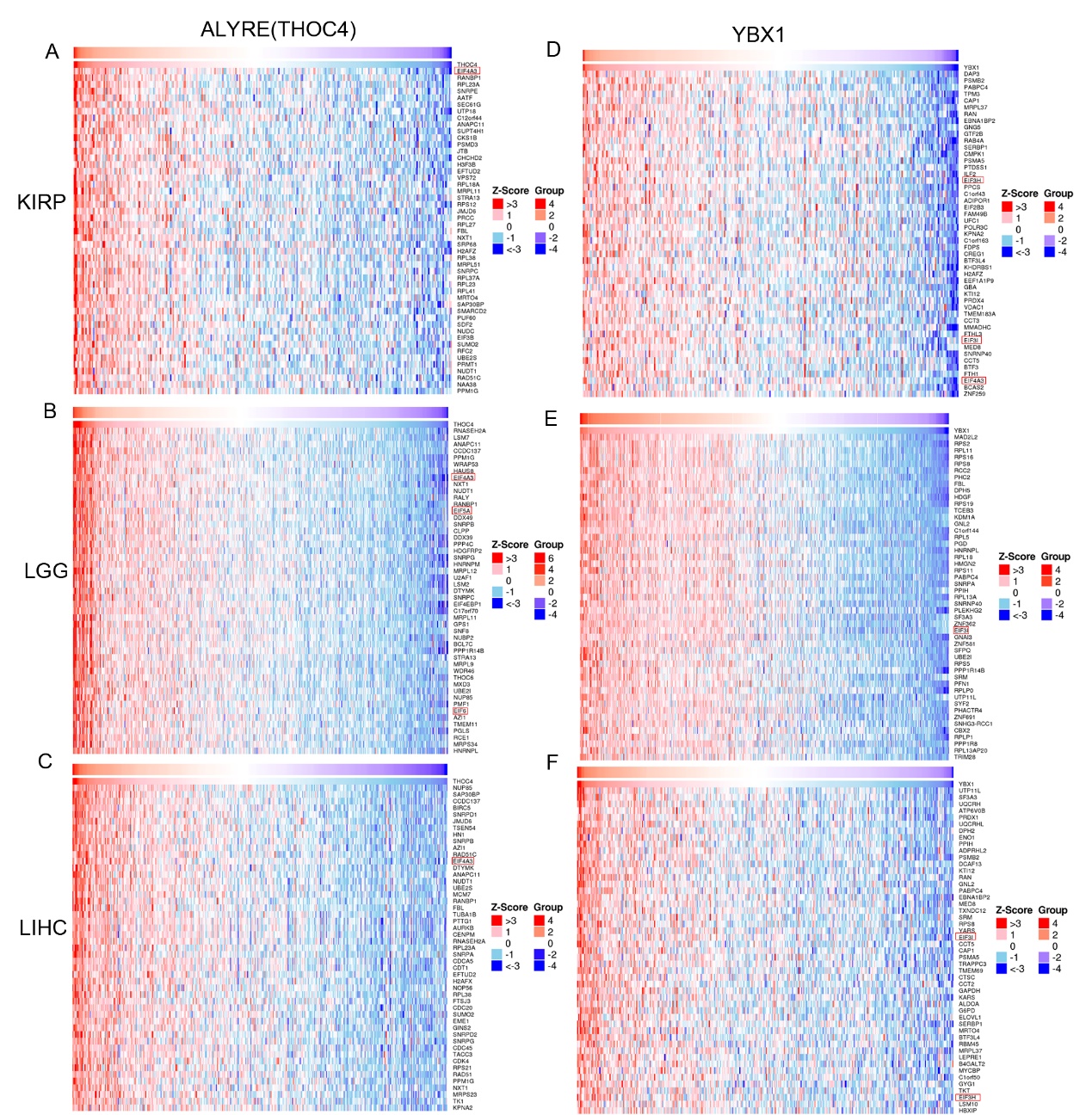


Figure S3. DEGs of m5C-reader-associated genes. (A-F) The top-fifty DEGs of (A-C) ALYREF and (D-F) YBX1 associated genes in KIRP, LGG and LIHC.


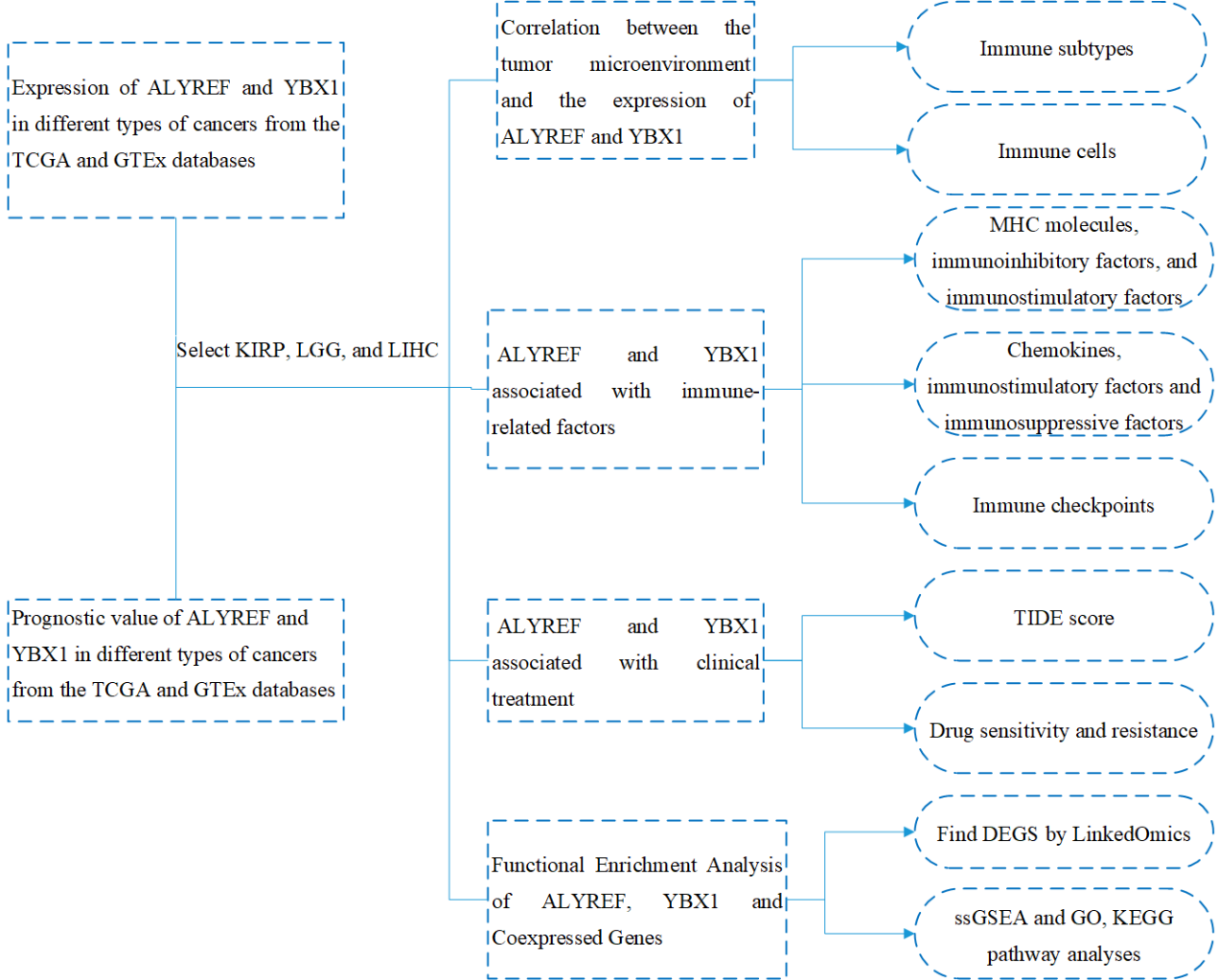


Figure S4. Workflow diagram for the study.
